# Correlation between adolescent chronic emotional stress and incidence of adult cardiovascular disease in female rats 

**DOI:** 10.22038/ijbms.2019.32888.7857

**Published:** 2019-10

**Authors:** Monireh-Sadat Mousavi, Alireza Imani, Sogol Meknatkhah, Gholamhossein Riazi

**Affiliations:** 1 Laboratory of Neuro-Organic Chemistry, Institute of Biochemistry and Biophysics, University of Tehran, Tehran, Iran; 2 Department of Physiology, School of Medicine, Tehran University of Medical Sciences, Tehran, Iran

**Keywords:** Cardiovascular disease, Emotional stress, Heart rate, M-Mode echocardiography, Natriuretic peptide receptor 3

## Abstract

**Objective(s):**

Association of adolescent emotional stress (ES) with the incidence of cardiovascular disease (CVD) at older age was investigated.

**Materials and Methods:**

21 female rats were divided into three groups of 7 each; ES, foot-shock, and control. Chronic ES was induced by exposing the rats to witness foot-shock of their neighboring counterparts in the stress-box system in 5 successive days. 6 weeks after the last stress exposure, M-Mode echocardiographic assessment, qRT-PCR, and western blotting were performed in adult rats to determine the persistent effect of adolescent ES on cardiac performance and gene/protein expression levels of cardiac natriuretic peptide receptor 3 (NPR3) as a biomarker of CVD.

**Results:**

Interventricular septum thicknesses in diastole (IVSd) increased from 0.152±0.007 cm to 0.197±0.016 cm (*P*<0.05), left ventricular posterior wall thickness in diastole (LVPWd) significantly enlarged from 0.169±0.006 cm to 0.288±0.033 cm (*P*<0.01), left ventricular posterior wall thickness in systole (LVPWs) enlarged from 0.223±0.012 cm to 0.318±0.038 cm (*P*<0.05), left ventricular mass increased from 1.000±0.024 g to 1.283±0.084 g (*P*<0.01), and mean heart rate elevated from 229.42±6.57 bpm to 280.29±10.45 bpm (*P*<0.01). Moreover, ES significantly upregulated the expression levels of cardiac NPR3 gene (*P*<0.01) and protein (*P*<0.01).

**Conclusion:**

The incidence of adult CVD seemed to be increased under the influence of adolescent ES. Consequently, we suggest that mental healthcare during adolescence would be a critical factor for adult CVD prevention.

## Introduction

Cardiovascular disease (CVD) is the first leading cause of death worldwide (1). Association between stress and CVD has been reported by numerous studies (2, 3). Since brain discriminates each form of stress through stimulating different neural networks and biochemical pathways, diverse forms of stress affect differentially on general health (4). Stress-induced physiological changes have also shown significant sex differences presenting females more vulnerable (5). 

A number of negative effects of psychological stress on cardiovascular performance have been reported. Pulmonary embolism (6), myocardial perfusion abnormalities (7), left ventricular dysfunction (8) and arrhythmia (9) are some reported disorders in response to short-term experimental stressors such as earthquake, acute mental stress, death in the family, court appearance and so on. However, the association of early-life emotional stress (ES) on the incidence of CVD in later age period has not been completely clarified. Therefore, we conducted an experiment to explore the long-lasting effect of adolescent ES on the incidence of adult cardiac abnormalities in female rats. Different cardiac structural and functional abnormalities are associated with increased CVD and other cardiovascular disorders, having their own risk for cardiovascular mortality. For example, increased left ventricular mass and thickness raise the risk of cardiovascular morbidity and mortality at least by 2-4-fold (10-12). Furthermore, we aimed to assess gene/protein expression of adult cardiac natriuretic peptide receptor 3 (NPR3) under the influence of adolescent ES. NPR3 is an essential cell-surface protein, which is expressed in the vasculature and is involved in vascular remodeling. A growing body of evidence demonstrates that NPR3, as a clearance receptor, thorough determining the circulatory levels of natriuretic peptides, modulates the physiological effects of whole natriuretic peptide system in the body (13, 14). This receptor is one of the novel candidate markers for CVD, which has been reported to be highly expressed in diverse cardiovascular disorders such as atherosclerosis (15), hypertension (16), cardiac hypertrophy (17), and heart failure (18). Moreover, the role of this protein has been progressing from diagnostic tool to therapeutic modality in recent years (14).

To achieve our objectives, a two-compartment box (stress-box) was applied for induction of ES to the experimental rats (19, 20). According to earlier studies on the effects of chronic stress on males and female rats, considerable sex differences have been observed in stress-induced neurobiological changes expressing females with more susceptibility (5, 21). Therefore, female rats were used for the present study, in spite of difficulties related to fluctuations in gonadal hormones during the estrous cycle. ES was induced by exposing the rats to witness electric foot-shock of their neighboring counterpart in the stress-box system. M-Mode echocardiography data acquisition in adult rats provided a rich picture of long-term effects of ES on cardiac parameters in respect to regional structural and functional changes. Moreover, evaluation of gene/protein expression levels of cardiac NPR3 in adult rats was beneficial to measure the severity of cardiovascular disorders. 

## Materials and Methods

***Animal***

Twenty-one female albino Wistar rats were obtained from the animal house of University of Tehran (Iran, Tehran). The rats were housed in a 12 hr day/12 hr night cycle (7.00 AM/7.00 PM) and temperature-controlled room (21-24 ^ᵒ^C), with free access to standard food and water. The experimental procedures were conducted based on the institutional guidelines of University of Tehran (Tehran, Iran). Maximum efforts were done to decrease the number of animals used and their suffering. 

***Rat estrous cycle phases determination***

In order to minimize the effect of female rat’s gonadal hormones on physiological parameters, the estrous cycle phases of the animals should be synchronized. To attain this, during the whole study, a mature male rat within a small cage was placed in each female rat’s cage with no mating possibility. Male urinary pheromones are known to synchronize the female rat’s estrous cycle phases (22). Prior to echocardiography, estrous cycle phases of all rats were determined by application of vaginal smear cytology according to protocol proposed by Marcondes in 2001 (23). In order to ensure identical phase for testing, the rats with different phase were subjected to echocardiography a few hrs earlier or later. The same procedure was carried out before decapitation. 

***Experimental design***

**Figure 1:**
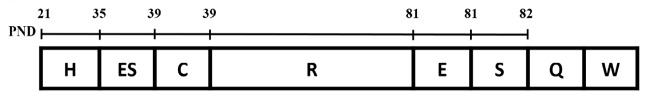
Schematic diagram of the experimental design. Two weeks habituation (PND 21–35); ES induction (PND 35–39); Serum corticosterone measurement (PND 39); Six weeks housing without stress (PND 39-81); 3-D echocardiographic assessment (PND 81); Sacrifice of experimental rats (PND 82); Quantitative PCR for cardiac NPR3; Western blotting for cardiac NPR3. H: Habituation, ES: Emotional Stress, C: Corticosterone, R: rest, E: Echocardiography, Q: Quantitative PCR, W: Western Blotting, PND: Postnatal day NPR3: Natriuretic peptide receptor 3

On postnatal day 21 (PND 21), the animals were randomly divided into three groups (7 rats each) of ES, foot-shock, and control. After two weeks habituation, the experimental group was exposed to ES in 5 successive days (PND 35–39); serum corticosterone was examined immediately following the last stress exposure to confirm stress induction (PND 39). After housing the rats for 6 weeks in their home cage (till PND 81), the echocardiographic study was performed in adult rats (PND 81) to evaluate long-lasting effects of adolescent ES on cardiac structural and functional changes. On the following day (PND 82), the rats were sacrificed by decapitation to determine the persistent effect of adolescent ES on gene/protein expression levels of cardiac NPR3 by qRT-PCR and western blotting (Figure 1).

***ES induction***

**Figure 2:**
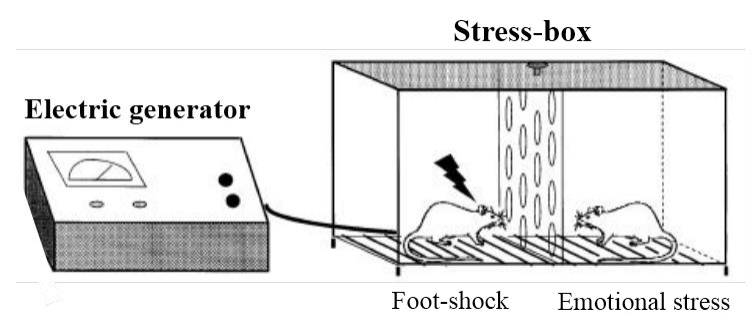
Schematic drawing of stress-box used for emotional stress induction. While one rat is shocked in the left chamber, the emotional stress was induced to other rat in the right insulated chamber through visual, auditory and olfactory contacts

A stress-box (30 cm × 25 cm × 30 cm) with stainless steel rods on the floor and two similar chambers separated by perforated transparent partition was utilized for ES induction (19, 20). Animal of ES and foot-shock groups were placed pairwise in the stress-box for a period of 10 min in 5 successive days (9 AM-12 noon). One chamber’s floor was connected to an electric generator (Tajhiz Gostar Omide Iranian CO. Iran) to apply foot-shock (0.25 mA, 50 Hz, 1 sec-duration, 10 shocks in 10 min-period with disordered intervals); however, the other chamber provided for ES induction was insulated. ES was induced by perceiving behavioral responses and emotional impressions (through visual, auditory and olfactory hints) from their counterparts subjecting to foot-shock in the adjacent chamber (Figure 2). Control animals were only placed in the stress-box system without any electric foot-shock or ES in the same period of time. The box was thoroughly cleaned with 70% ethanol after each trial. 

***Serum corticosterone measurement***

Blood samples were collected from the rats lateral tail veins, and the serums were immediately separated. Serum corticosterone was measured in triplicate aliquots by a commercial ELISA kit (DRG international, USA; Cat. No. EIA-5186) according to the manufacturer’s guidelines. The detection limit of the assay was 4.1 ng/ml. The intra-assay coefficient of variation (CV) ranged from 2.8 to 8.3% with a mean of 5.3% and inter-assay CV ranged from 4.8 to 12.4% with a mean of 8.2%.

***Echocardiography***

In PND 81, the echocardiographic study was performed according to the method of Tousi *et al* (24). First, all the rats were anesthetized with a mixture of ketamine and xylazine [80+8 mg/kg, IP] (25). Anesthetized animals were placed on the desk in the supine position. Their chest hair was shaved. The ultrasound gel was rubbed on the thorax area for placing the probe of echocardiogram on it. M-Mode echocardiography data acquisition was performed with an echocardiographic system (GE Voluson 730 Pro, Kretztechnik Company, Austria). The average of 3 consecutive cardiac cycles was considered the meaningful data. M-mode tracings using MATLAB R2011b software were obtained to determine the echocardiographic parameters: interventricular septum thicknesses in diastole (IVSd); left ventricular diameter in diastole (LVDd); left ventricular posterior wall thickness in diastole (LVPWd); interventricular septum thicknesses in systole (IVSs); left ventricular diameter in systole (LVDs); left ventricular posterior wall thickness in systole (LVPWs); left ventricular ejection fraction (LVEF); fractional shortening (FS). Using the special formulas (mentioned in Table 1) and some above-mentioned parameters, more cardiac parameters (diastolic volume (DV); systolic volume (SV); stroke volume; cardiac output (CO); left ventricular mass (LV-mass)) were calculated (26, 27).

**Figure 3:**
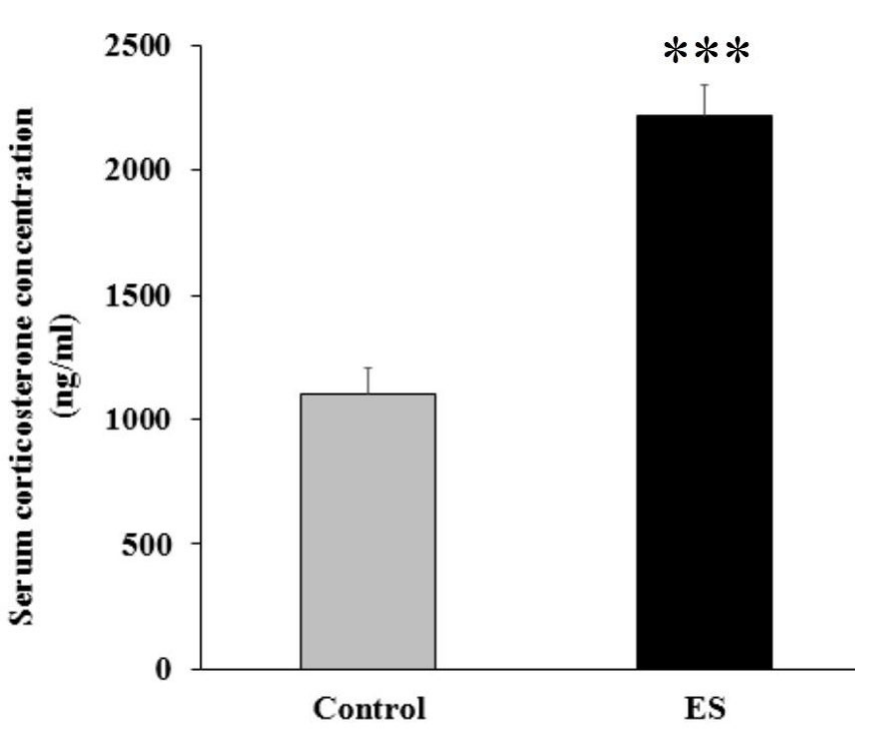
Serum corticosterone concentration. Emotionally stressed-rats exhibited significant increase in serum corticosterone concentration more than two-fold. All data are expressed as mean±SEM. Data were analyzed by two-tailed unpaired t-test. *** *P*<0.0001

**Figure 4:**
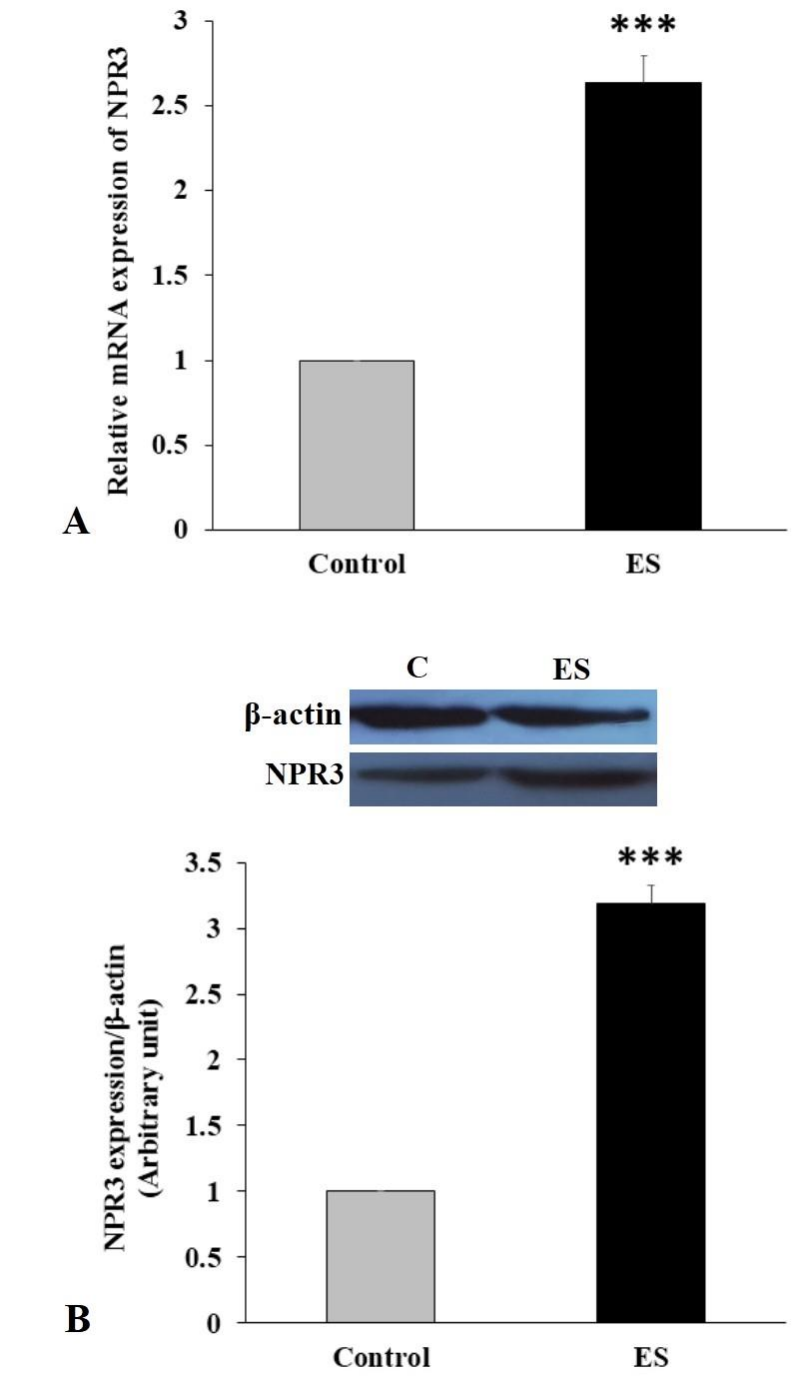
Long-term effects of ES on gene/protein expression levels of cardiac NPR3 in female rats. (A) qPCR (N=4 per group) and (B) Western blotting (N=3 per group) for cardiac NPR3 exhibited upregulation of cardiac NPR3 mRNA and protein. All data are presented as mean ± SEM. Data were analyzed by two-tailed unpaired t-test. ES: Emotional stress; qPCR: Quantitative PCR; NPR3: Natriuretic peptide receptor 3. *** *P*<0.001

**Table 1:** Equations for deriving echocardiographic parameters

**Parameter**	**Equation**
**DV, ml**	1.047 (LVDd)^3^
**SV, ml **	1.047 (LVDs)^3^
**STROKE VOLUME, ml**	DV-SV
**CO, ml/min**	STROKE VOLUME × HEART RATE
**LV-mass, g**	1.04 [(LVDd + LVPWd + IVSd)^3^-LVDd^3^] × 0.8+ 0.6

***Abbreviations=*** **DV:** diastolic volume; **SV:** systolic volume; **CO:** cardiac output;** LV-mass:** left ventricular mass; **LVDd:** left ventricular diameter in diastole; **LVDs:** left ventricular diameter in systole; **LVPWd:** left ventricular posterior wall thickness in diastole; **IVSd:** interventricular septum thicknesses in diastole.

**Table 2:** The sequence of primers used in qPCR

**Gene name**	**Primer sequence**
**NPR3**	Forward: 5'-GGCCGGTTCAAAATGCGA-3' Reverse: 5'-GGCCATTAGCAAACCAGCAC-3'
**HPRT**	Forward: 5'-CTCATGGACTGATTATGGACAGGAC-3' Reverse: 5'-GCAGGTCAGCAAAGAACTTATAGCC-3'

**Table 3:** Echocardiographic parameters

**Parameter means ** **± SEM**	**Control**	**ES**	***P*** **-value**
**IVSd, cm**	0.152 ± 0.007	0.197 ± 0.016 ^*^	0.0188
**LVDd, cm **	0.533 ± 0.016	0.487 ± 0.030	0.1798
**LVPWd, cm **	0.169 ± 0.006	0.288 ± 0.033 ^**^	0.0016
**IVSs, cm**	0.256 ± 0.009	0.280 ± 0.016	0.1862
**LVDs, cm**	0.287 ± 0.025	0.261 ± 0.033	0.5279
**LVPWs, cm **	0.223 ± 0.012	0.318 ± 0.038 ^*^	0.0148
**HR, bpm**	229.42 ± 6.57	280.29 ± 10.45 ^**^	0.0011
**LVEF, %**	81.750 ± 3.353	83.167 ± 2.701	0.7604
**FS, %**	46.625 ± 3.868	48.333 ± 4.248	0.7730
**DV, ml**	0.162 ± 0.016	0.128 ± 0.022	0.2374
**SV, ml**	0.029 ± 0.007	0.023 ± 0.006	0.5242
**Stroke volume, ml**	0.133 ± 0.012	0.106 ± 0.017	0.1933
**CO, ml/min**	30.108 ± 1.943	27.095 ± 4.236	0.4782
**LV-mass, g**	1.000 ± 0.024	1.283 ± 0.084 ^**^	0.0031

***Abbreviations=*** **IVSd:** interventricular septum thicknesses in diastole; **LVDd:** left ventricular diameter in diastole; **LVPWd:** left ventricular posterior wall thickness in diastole; **IVSs:** interventricular septum thicknesses in systole; **LVDs:** left ventricular diameter in systole; **LVPWs:** left ventricular posterior wall thickness in systole; **HR:** heart rate; **bpm:** beat per min; **LVEF: **left ventricular ejection fraction; **FS:** fractional shortening; **DV:** diastolic volume; **SV:** systolic volume; **CO: **cardiac output; **LV-mass:** left ventricular mass.

^*, **^ represented significant difference from control (*P*<0.05 and *P*<0.01, respectively).

***Decapitation and sampling***

After echocardiographic assessment, the rats were sacrificed by decapitation on the following day (PND 82), and heart tissue samples were immediately removed and rinsed in phosphate-buffered saline (PBS). The samples were then snap frozen in liquid nitrogen and stored at -80 ^°^C in two groups separately for qPCR and western blotting. 

***Quantitative PCR (qPCR)***

Quantitative PCR was performed according to the method of Parsa *et al.* with minor modifications (28). Total RNA was extracted from frozen heart tissues using TRIzol (Invitrogen, Carlsbad, CA). Then, complementary DNA (cDNA) was synthesized using a Prime Script RT reagent kit (Takara, Cat RR037A). The expression levels of NPR3 gene were measured by qPCR Master Mix for SYBER Permix Ex Taq (Takara, Cat.RR280L) and quantified using the Rotor-Gene 6000 (Qiagen). All reactions were performed in triplicates. Hypoxanthine phosphoribosyltransferase-encoding gene (HPRT) was used as a housekeeping gene (28). Finally, the expression level of NPR3 gene was normalized against HPRT. The primer sequences are listed in Table 2.

***Western blotting***

Protein extraction and western blot analysis was performed according to the method of Mard *et al* (29). Total protein was extracted from frozen heart tissues using RIPA (Radioimmunoprecipitation assay) buffer containing 25 mM Tris-HCl pH 7.4, Triton X-100 1% or NP-40 1%, sodium dodecyl sulfate (SDS) 0.1%, sodium deoxycholate 0.5%, 150 mM NaCl, 1 mM EDTA and dd-H_2_O in addition to complete mini protease inhibitor cocktail (Roche, Indianapolis, IN, USA). The extracted proteins were separated by SDS-PAGE on 10% acrylamide gels and transferred onto nitrocellulose membranes. The membranes were immersed for 6 hrs in Tris-buffered saline with 0.1% Tween 20 and 5% non-fat dry milk as blocking reagent (TBST, pH: 7.6). The blocked membranes were then incubated overnight at 4 ^°^C with the anti-NPR3 antibody (ab14355, Abcam, USA), or anti-β-actin antibody (ab20272, Abcam, USA). On the following day, membranes were washed 5 times with TBST and then incubated with a rabbit polyclonal secondary antibody to mouse IgG HRP for 90 min at room temperature. Finally, a chemiluminescence western blotting system was used to detect labeled proteins. All reactions were performed in triplicates. The expression level of NPR3 protein was quantified by Image J software and the values were normalized to β-actin as a housekeeping protein. 

***Statistics***

Statistical analysis was performed with the GraphPad InStat 3 statistical package (GraphPad InStat Software, San Diego, CA). Data were analyzed using two-tailed unpaired t-test and expressed as mean ± SEM. *P*-values less than 0.05 were considered statistically significant differences between the groups. 

## Results

***Serum corticosterone concentration***

Serum corticosterone concentration was increased by more than two-fold (from 1104.8±100.12 to 2212.4±131.44) in response to ES. The significant elevated corticosterone level verified proper induction of stress (*P* <0.0001; Figure 3).

***Echocardiography***

The effects of ES on echocardiographic parameters are summarized in Table 3. Two-tailed unpaired t-test on echocardiographic data represented significant increase in left ventricular mass and geometry; the mean IVSd increased from 0.152±0.007 cm to 0.197±0.016 cm (*P*<0.05), the mean LVPWd increased from 0.169± 0.006 cm to 0.288±0.033 cm (*P*<0.01), the mean LVPWs increased from 0.223±0.012 cm to 0.318±0.038 cm (*P*<0.05), and the mean LV-mass increased from 1.000±0.024 g to 1.283±0.084 g (*P*<0.01). Moreover, the mean heart rate elevated from 229.42±6.57 bpm to 280.29±10.45 bpm (*P*<0.01). No considerable change was observed in other echocardiographic parameters across the experimental and control groups.

***qPCR and western blotting analysis***

According to statistical analysis, adolescent ES had significant effects on gene/protein expression levels of cardiac NPR3 at older age. As shown in Figure 4, the level of mRNA and protein expression of cardiac NPR3 in ES group were extremely higher in comparison with control group (*P*<0.001).

## Discussion

A strong inverse relationship between stress and cardiovascular performance has been reported by several studies (30, 31). Stress could be classified to acute and chronic, in terms of time (32), and to physical and emotional, in terms of nature (33). Although the negative effects of ES on cardiovascular health has been identified (34, 35), the link between early-life ES and incidence of CVD at older age has not been clearly determined. Therefore, we investigated the long-lasting effects of adolescent chronic ES on adult echocardiographic parameters and gene/protein expression levels of cardiac NPR3, as a biomarker of CVD in female rats. 

After the last stress exposure, emotionally stressed-rats displayed a drastic increase in serum corticosterone concentration compared to their control counterparts. This result verified proper stress induction (36). Based on the earlier studies, corticosterone concentration not only depends on the level of stress exposure, but also on sex, phases of the estrous cycle and, most importantly, diurnal rhythm (37). Thus, a relatively high level of serum corticosterone is the consequence of late afternoon blood sampling during proestrus phase of female rats.

According to the results, the considerable increase in heart rate and left ventricular mass/geometry demonstrated sympathetic nervous system (SNS) hyperactivity and left ventricular hypertrophy induced by ES. Earlier research studies have reported a 2-4-fold risk of cardiovascular morbidity and mortality in patients with left ventricular hypertrophy compared to those with normal left ventricular mass and thickness (38). Proinflammatory cytokine levels, as indicators of susceptibility to disease, are increased in response to elevated heart rate (39, 40), abnormal left ventricular mass/ geometry (41, 42), and sustained SNS hyperactivity (43). As a result, it appears that adolescent ES by increasing the risk of left ventricular hypertrophy and elevated heart rate not only increases the cardiovascular mortality but also increases the susceptibility to other diseases and disorders.

Moreover, as shown in the present study, the gene/protein expression of cardiac NPR3 in emotionally stressed-rats was significantly more than that in non-stressed rats. The higher the expression of cardiac NPR3 is, the more the incidence of CVD will be (44). Therefore, our results validated the key role of adolescent emotional health in adult cardiovascular performance (45, 46). Our findings are in accordance with our earlier studies showing the harmful effects of adolescent repeated ES on multiple sclerosis pathology (47), cognitive performance and oxidative stress (48) in adult female rats.

## Conclusion

Based upon the results of the study, under the influence of adolescent ES, the incidence of adult CVD seemed to be augmented with increased left ventricular mass/geometry, heart rate, and cardiac NPR3 gene/protein. It can be concluded that mental health care during adolescence would be a critical factor in adult CVD prevention. It is recommended for future research to consider the histological and molecular approaches towards clarifying mechanisms involved in the long-term effect of ES on cardiovascular performance.
